# Assessing the policy and practice impact of an international policy initiative: the *State of the World’s Midwifery 2014*

**DOI:** 10.1186/s12913-018-3294-4

**Published:** 2018-06-27

**Authors:** Kathryn Oliver, Zachary Parolin

**Affiliations:** 1 Department of Social and Environmental Health Research, London School of Hygiene and Tropical Medicine, London, UK; 2 Herman Deleeck Centre for Social Policy, University of Antwerp, Antwerpen, Belgium

**Keywords:** Policy evaluation policy impact, Policy change, Public health, Midwifery

## Abstract

**Background:**

Understanding how policies lead to changes in health systems and in practice helps policymakers and researchers to intervene more successfully. Yet identifying all the possible changes that occur as a result of a new policy is challenging not only methodologically and logistically, as limited resources are available to conduct indefinite evaluations, but also theoretically, as a complete mapping and attribution of post-hoc changes requires a full understanding of the mechanisms underpinning all change. One option is to identify possible changes across a number of policy impact domains.

**Methods:**

Using a Policy Impact Framework, we brought together data from media, documents and interviews to identify changes to midwifery policy, practice and provision, following the launch of a new global policy initiative, the State of the World’s Midwifery (SoWMy 2014) report published in 2014. We used these identified impacts to develop a map of the mechanisms underpinning these changes.

**Results:**

SoWMy 2014 contributed to a number of changes at national levels, including increased status of midwifery within national governments, improved curricula and training opportunities for midwives, and improved provision of and access to midwifery-led care. These contributions were attributed to SoWMy 2014 via mechanisms such as stakeholder interaction and acquisition of government support, holding national and international dissemination and training events, and a perceived global momentum around supporting midwifery provision. Policy initiatives of this kind can lead to changes in national and international policy dialogue and practice. We identify factors and mechanisms that are likely to increase the scope and scale of these changes, at contextual, national and global levels.

**Conclusions:**

Identifying changes following a policy using a policy impact framework can help researchers and policymakers understand why policies have the effect they do. This is important information for those wishing to increase the effectiveness of future policies and interventions.

**Electronic supplementary material:**

The online version of this article (10.1186/s12913-018-3294-4) contains supplementary material, which is available to authorized users.

## Background

Policy impact assessment aims to identify potential (ex-ante) and actual (ex-post) changes which occur following the introduction of a policy [1]. Policy impact assessment has become part of the normal practice of global and national policymaking [2]. Yet, as Adelle identifies, despite increasing diversity in assessment methodology (see, e.g. [3]), the theories underpinning these methodologies are less well-articulated. For example, many impact assessments rely on a linear perception of the policy process, which has largely been discredited by current policy scholars [4].

Rather, policy leads to changes in unexpected ways, in unanticipated domains, due to the complex and messy nature of policy making and implementation [5]. There are many proposed methods to evaluate impact of research on policy [6] such as the Payback model [7], originally used in health but now applied across policy domains. These models often attempt to quantify impact using quantitative metrics, such as citations, and are therefore not well able to cope with the complex or unexpected. One approach which does try and do this is contribution mapping [8, 9], in which a new intervention, policy or piece of research is followed over time, and impacts are identified through interviews, documentary analysis, or other multi-modal means. This has the advantage of not pre-specifying the outcomes, and allows for flexibility in evaluative methods. We can also capture people’s perceptions and experiences, and how those changed as a result of the intervention; and these can play a vital role in any mechanism underpinning real world change. In this paper, we use this broad and holistic approach to consider all the contributions made by a global policy initiative, the State of the World’s Midwifery 2014 (SoWMy 2014, [10]).

## Methods

### Aim, design and setting

SowMy 2014 constitutes a policy initiative; taking action on behalf or, or through global actors to achieve policy change in a specific area – in this case, midwifery services. Our aim, therefore was to employ a ‘policy impact evaluation framework’ developed by Oliver [11], to map identified changes against different domains, such as behaviour change, or changes to policy and practice. This framework was developed through an analysis of policy impact case studies submitted to the Research Excellence Framework in the UK in 2014. We used these case studies to develop a typology of policy impacts (i.e. domains on which policy may case changes). In this report, we use this framework to explore the types of contributions which SoWMy 2014 made, and to interrogate possible mechanisms which underpin these changes. We synthesise these data into a Theory of Change which allows us to draw generalisable lessons about how policy initiatives may be best supported to create and accelerate changes in policy and practice.

#### Policy impact framework

To assess the contributions of an international policy initiative is challenging, as changes may occur at different levels (international/national/regional/local), in different time scales (days to decades), and across a wide range of domains (attitudes, behaviours, resources, policies and practices, and so on). Collecting information about all of these changes requires a broad and holistic approach. It is not realistic to attempt an exhaustive mapping across each of these axes; instead, indicative sampling can be used to demonstrate the range of impacts and mechanisms leading to these impacts.

We identified a number of domains in which changes could occur. This framework allows impacts to be gathered and organised into a pre-existing set of categories that contribute to our understanding of change mechanisms (see Table 1). We used these categories to map data on policy impacts, allowing us to build up a picture of the kinds of contributions made and triangulate between the data sources. Using this framework, we developed a theory of change which allows us to develop understandings of the mechanisms leading to policy change.

**Table 1:** Policy impact framework (adapted from [11])

● Who is impacted ● Changing opinions/attitudes ● Production of new knowledge ● Production of concrete tangible outputs ● Creating capacity and skills-building ● Changing procedures/practice/internal policies	● Change or influence on policy/government. ● Interactions with stakeholders ● Changes to behaviour ● Changes to social environment ● Changes to physical environment ● Changes to reputation and esteem

For these reasons, we have used a range of methods to identify possible changes; semi-structured interviews and written interviews with policymakers and practitioners associated with the development and launch of the initiative, media and social media coverage, and academic and policy documents referencing the findings of SoWMy 2014. Below, we describe in detail each of these sets of data, and how we brought them together using a common analytical framework.

### Data collection

#### Media and social media coverage

To capture media coverage of and community responses to SoWMy 2014, we collected data and utilised prior reports on media coverage around the policy initiative. We examined the number of news stories, countries of origin and types of output (feature, news, analysis) which related to SoWMy 2014. We searched available electronic databases for citations, using search terms developed from the SoWMy report. As part of this search we identified a previously-published media analysis of the SoWMy initiative [12] which collated media reports and provided a comprehensive overview of media attention during the month following the global launch. We extended Edelman’s search methods to include July 2014 to January 2016 to draw a more complete picture of SoWMy’s media impact in the 18 months following its global launch. We included reports from all publishers and organisations, and included all languages which we could interpret (French, German, English, Dutch, Spanish and Danish).

We also tracked social engagement metrics on Twitter and Facebook to estimate how widely the SoWMy 2014 reports had been shared, as well as the types of responses and organisations / individuals engaging with the disseminations. Finally, we also attempted to identify relevant blogs through brief Internet searches.

#### Academic citations

To capture concrete policy responses and academic evaluations / responses to SoWMy, we sought documents relating to the SoWMy 2014 report. We used Google Scholar and Internet databases to identify documents citing the SoWMy 2014 report, and also examined those citing the earlier 2011 report for relevance. We collected numbers of citations, details about published reports, and numbers of academic papers written from or about SoWMy.

#### Semi-structured interviews

Semi-structured and written interviews were conducted to analyse how the SoWMy report was perceived and utilised in the 18 months following the introduction of the global report. All interviews were conducted by one of the authors (Parolin) who collated field notes after each interview.

To identify participants for the interviews, 72 potential respondents were first selected purposively from a list of approximately 900 individuals who in some way contributed to the 2014 SoWMy report. The potential respondents were selected to represent the geographical range within which SoWMy 2014 was conducted, in consultation with partners. Each potential respondent was initially contacted via email and was provided an information sheet that outlined the purpose of the evaluation. Additionally, each respondent who agreed to participate was asked to sign a consent form prior to the interview or completion of the questionnaire. The consent form outlined the participant’s rights and acknowledged that any insights provided would be collected and used anonymously for the purpose of this evaluation.

A topic guide was used to guide the open-ended interview during the 11 phone or Skype conversations (see Additional file 1). The topic guide was designed to gain the following information from the respondents, listed here in broad terms:

- Respondent’s professional responsibilities and involvement with the SoWMy report
- Respondent’s perception of:
  ○ SoWMy report’s local and global goals, as well as perceived challenges in achieving desired results
  ○ Policy/programmatic changes that have occurred since launch of the report, and what role, if any, the report played in influencing such changes
  ○ Specific ways in which the SoWMy report (including local versions in applicable countries) has been utilized since its release
  ○ Key challenges in achieving local or national ambitions relating to midwifery and Sexual, Reproductive, Maternal and Newborn Health (SRMNH) Services
  ○ How the development and implementation of SoWMy could have been improved

Questionnaire forms were structured in the same format and order as the topic guides and were designed to garner the same insights.

All questionnaire forms and audio recordings from Skype and phone conversations were securely stored after their completion. The audio recordings were transcribed through an independent contractor, after which all original recordings were deleted from the recording device. Ethics approval was sought and granted from Oxford University (Ref: SSH_SPI_C1A_15_006).

The authors analysed transcripts using a mixed coding method. This approach combined elements of a priori coding, which allowed prior literature on program evaluation to suggest potential themes that might emerge, and grounded coding, which allowed for the possibility that unexpected themes might emerge from participant responses. Both authors discussed the interviews regularly to assess emerging findings. Once all were completed, both authors conducted preliminary thematic coding of the transcripts, which were compared for similarities. We used these conversations to develop a common understanding about the domains within the policy impact framework, and ZP populated this subsequently. We also used these conversations to develop hypotheses about mechanisms, for which we sought data from the interviews and other data sources. We utilised thematic mapping techniques to organize participant responses into common topics, such as *outcomes of the SoWMy report* (working groups, training education, and more), *factors influencing response to SoWMy launch* (media coverage, advocacy toolkit, and more), as well as several other themes.

## Results

### Media data

Data on the distribution of SoWMy-related stories and mentions were collected to assess the reach of the report after its national launch, exploring quantitative metrics on frequency of distribution, blog posts and academic articles.

We identified approximately 1000 public media stories or press releases mentioning SoWMy 2014, of which the majority were published within the first month after launch. Independent coverage spanned a wide range of countries and included high-visibility outlets such as Time, Reuters, Huffington Post, The Guardian, and any more. Additionally, the press release introduced after the global report’s launch was also picked up more than 300 times across a range of digital outlets. In addition, we identified 11,330 social media accounts mentioning SoWMy (predominantly within the same timeframe), 10 blog posts, and an existing media analysis of the report [12].

From July 2014 onward, media reports and digital mentions reduced significantly – down to an average of 164 mentions per month July to December 2014, for example, and down to fewer than 5 per month by January 2015. A brief spike in social media activity occurred during the Nigerian national launch in November 2014; aside from this event, digital mentions and blog posts were largely limited to the month following the national launch of the SoWMy report.

### Academic and research citations

Using Google Scholar and Scopus, an online academic database, 30 academic and scientific articles referencing or referring to the 2014 SoWMy Report were identified, an increase on the 15 citations garnered by the SowMy 2011 report. Most of these articles were published in 2015 and were presented in a variety of professional journals and publications, including *The Lancet*, *Journal of Midwifery & Women’s Health*, *Tropical Medicine & International Health*, and *Midwifery.* These articles focused on a range of topics, including identification of local maternity mortality [13–17], introduction of or assessment of feasibility of changes to midwifery training and education [18–21] or methods to reduce stillbirths [22–24]. A significant proportion of these articles were commentaries on the provision of or policies around midwifery at national and global levels (see [20, 22, 25–28]). No current publications, though, provide empirical analyses of the development, implementation or effectiveness of the SoWMy initiative.

Additionally, the SoWMy report launch date was related to the timing of *The Lancet’s* midwifery series, which included 10 research publications on the topic of midwifery.

### Interview data

Of the 72 respondents contacted, 19 agreed to participate (a success rate of 26%) and completed either a Skype interview or a questionnaire form between December 2015 and February 2016. As outlined in Table 2 below, the 19 respondents reflected a range of geographical regions, organizational types, forms of involvement with the report, and personal characteristics.

**Table 2:** Characteristics of Respondents Interviewed for Evaluation

Sex	Geographic Region	Organisation Type	Relation to SoWMy Report
14: Female 5: Male	6: Southeast Asia 6: Sub-Saharan Africa 3: Middle East / Northern Africa 1: East Asia 1: Latin America 1: North America 1: Western Europe	10: National UN Agency 5: Local Midwifery Associations 2: National Government Officials 2: Academic Bodies	12: National Involvement 5: Local Involvement 2: Global Steering Committee Members

Of the 19 respondents, 11 were able to complete the interview via phone or Skype, while eight requested to complete written questionnaire forms. Four individuals declined the invitation to participate, and 49 did not respond to interview requests. Among the four individuals declining to participate, two were on temporary leave, one had recently departed the office, and one cited lack of time.

Most respondents were engaged in assisting with data collection, providing policy advice, supporting midwifery training and education, or advocating on behalf of midwives and other SRMNH workers. Respondents discussed how they used, or did not use the SoWMy 2014 report, the impact, if any, of the report, and if relevant the ways in which they planned to use the report to achieve policy and practice impacts. These data, together with the media and citation data, are collated in the policy impact framework to follow.

### Policy impact framework

We organise our data using a policy impact framework to explore SoWMy’s particular contributions to midwifery-related efforts on the national and global levels. We then plot the data in an organised, visual format within the theory of change.

#### Who is impacted?

The data reveal that SoWMy impacted several groups of individuals, including policy makers (within government and otherwise), midwifery advocates and associations, and the midwives of the countries examined within the report. The mechanisms through which each of these groups is impacted varies. Midwifery advocates and interest groups were often the first set of individuals to receive and utilise the report. Using a variety of tactics (including events, localised reports, and political outreach), these individuals and groups contacted policymakers at national levels about SoWMy, aiming to achieve some level of policy or programmatic change, directly or indirectly affecting the country’s population of midwives.

#### Changing opinions/attitudes

Our data show that the report was often used to educate midwives, midwifery advocates, and government officials on relevant statistics related to midwifery care and maternal health within their respective countries – such as trends related to maternal or prenatal mortality, the frequency of family planning visits, gaps in midwifery workforce availability, geographic barriers to care access, and financial limitations to boosting the care workforce. This new information, which often came from the country-specific pages within the SoWMy 2014 report, formed or altered the opinions of many midwifery advocates and organisations on the necessary actions to be taken to improve maternal health on the local, regional, and national levels. Midwives and their advocates frequently reported using the report and its statistics as an educational tool to highlight challenges and influence the opinions and decisions of policymakers and government officials.

“[The report] contributed to improve the knowledge of the decision makers, health managers and health workers on the current situation of midwives in Viet Nam, and their important roles in the obstetric and newborn care.” (National Government Official, Viet Nam).

#### Production of new knowledge

The SoWMy report served an impetus for the development of new research and the creation of new reports on national midwifery progress. As noted previously, approximately 30 academic publications have referenced the findings of SoWMy 2014 since its global launch. Additionally, according to nearly all of the respondents interviewed, the process of compiling the report itself generated new knowledge related to national midwifery trends and needs, and allowed benchmarking of progress to date against the global strategy for maternal health:

“[The report] clearly revealed our status… It helps us to know where we are and what exactly we are doing with regards to maternal health problems.” (Member of Local Midwifery Association, Liberia)

The SoWMy report was reported to lead to a number of national reports and events, often designed to supplement and localise the relevance of the findings for policymakers and government officials. In some cases, where the global report was perceived as too broad-brush or inaccurate at a national level, production of local versions were politically more palatable and more likely to lead to change. Local versions of the SoWMy report have been developed In Ethiopia, Mexico, Lao PDR, Morocco, Afghanistan, and Nigeria, among other countries.

#### Production of concrete tangible outputs

The most common tangible outputs, according to data collected from interview respondents, were locally-tailored versions of the SoWMy report. These localised versions came in different forms. In Lao PDR, for example, the country-specific information was extracted from the global report and simply translated into a local language to enhance readership and accessibility. In Mexico, however, the national report brought in new information and provided more in-depth analysis on national challenges. Regardless of particular approach, these reports were most often used to supplement, rather than substitute, the findings within the global SoWMy report. Other tangible outputs included curricula, guidelines and policies, as detailed below.

#### Creating capacity and skills-building

In multiple countries, SoWMy led directly to the creation of working groups and/or midwifery organisations, which enhanced the capacity of governments and advocacy groups to highlight and suggest specific policy or programmatic changes related to midwifery and maternal health. Lao PDR, for example, drafted and approved the constitution of a Midwifery Society in late 2015. In Mexico, SoWMy inspired the creation of a National Intersectorial Group to attempt to raise the number of midwifery schools and enhance education; in Morocco, a Basic Education Curricula was approved.

#### Changing procedures/practice/internal policies

Several countries reported changes to midwifery curricula and training materials to enhance the quality of education -- a key aim identified within the SoWMy report. Policies to support and standardise midwifery training were instituted in several countries, and midwifery recruitment and retention were reported to be set as a policy priority in the strategic plans of several ministries of health. As described previously, new working groups were also created to facilitate changes to midwifery practices in Mexico, Lao PDR, Morocco, and elsewhere.

#### Change or influence on policy/government

Governments were reported to make multiple changes following the SoWMy 2014 report, including: a commitment to increase the quantity of midwives and quality schools (Viet Nam, Morocco, Mexico, Nigeria, Myanmar, Lao PDR, and more); the formation of new working groups to coordinate policy changes (Mexico, Morocco, Lao PDR, and more); enhancement of curriculum and national training materials (Nigeria, Morocco, Viet Nam, and more); greater political awareness and prioritization of the needs of midwives (as nearly all respondents indicated); greater understanding of specific challenges related to maternal health (all); and greater collaboration between midwifery advocates and government officials.

#### Interactions with stakeholders

Many respondents indicated that the SoWMy 2014 report facilitated enhanced and more frequent correspondence with policymakers and other key stakeholders after the global launch. These interactions appear to be an important mechanism in gaining political support and placing the issues highlighted within the SoWMy report on the political agenda.

In particular, national and regional events and workshops were consistently highlighted as an effective mechanism by which to share findings and stimulate discussion with key stakeholders. Even among countries where no national report was introduced, the global report was often sufficient for advocates to garner the attention of and create dialogue with relevant government bodies.

#### Changes to behaviour

Changes to everyday behaviours following the SoWMy report were not reported or observed.

#### Changes to social environment

The strengthening of midwives’ standing within the broader health community was one outcome of the report. In particular, advocates aimed to leverage SoWMy 2014’s findings to advance the occupational prestige of the midwifery profession and, thus, to alter its relative standing in the social environment of the health services industry [29].

“The report was important in creating a united front in the nursing and the midwifery communities.” (Global Steering Committee Member).

Another respondent framed the strategy as an attempt to raise the profile of midwives and midwifery generally:

“One of the main aims and effects of the SoWMy 2014 report was to give visibility to the role of midwives in the health system.” (Member of Local Midwifery Association, Morocco; translated from French).

#### Changes to physical environment

Changes to physical environment following the SoWMy report were not reported or observed.

#### Changes to reputation and esteem

Enhancing the reputation and status of the midwifery profession was a common theme among interviews with respondents. Many claimed that bolstering such recognition was an important contribution of the SoWMy report, and some even deemed it a necessary step toward achieving progress with respect to midwifery-related goals. Many respondents felt that this enhanced status was a more common outcome than attributable, concrete policy change.

### Challenges

While SoWMy 2014 was clearly influential in stimulating changes to policies and programmes related to midwifery and SRMNH, respondents noted a number of challenges associated with the data collection and analysis for the report, with promotion and use of the report, and with the overall context within which the global strategy was developed and implemented.

#### Timing and length of data collection

The most frequent set of challenges concerned the data collection process during the development of the report. Some respondents felt that the overall process of data collection was too short, leading to incomplete or unsatisfactory data collection. The timeframe meant that it was difficult to mobilise resources effectively, as indicated by one respondent:

“You cannot plan, and you have reasons why it’s so difficult… The government doesn’t have this data ready. It’s not readily available, it’s also not transparent, and they don’t display it on any website, so you really have to dig into papers, literally.” (Member of National UN Agency, Southeast Asia)

Still, other respondents mentioned that the length of time between the data collection process and the release of the global report might have contributed to hesitancy of some political leaders to accept the report.

“During that time [between data collection and the launch of the global report], lots of things changed… with the following consequence: the government said, “Well, this is not right anymore,” and we had to say yes, but this is how we did the data collection.” (Member of National UN Agency, Southeast Asia).

#### Complexity

Multiple respondents commented on the complexity of the data questionnaire. This questionnaire was said by some to be too difficult or too long to easily complete.

“It could be simplified by far… they are so complicated and so in-depth, and some of it the countries don’t find relevant, and that’s quite demotivating in getting the data.” (Member of National UN Agency, Southeast Asia).

Another respondent added to the concern of complexity, noting that it was unclear how certain metrics within the report were calculated. This harmed the perceived authenticity of the results, according to the respondent.

#### Involvement of additional stakeholders

Finally, multiple respondents noted that the data collection process might have benefited from inclusion of other types of participants, such as local stakeholders, government officials, and, from a technical perspective, more statisticians and midwifery researchers.

Aside from data-related concerns, several respondents also noted that communications-related support after the global SoWMy launch could have abetted their efforts to utilise the report.

Other challenges that respondents brought up had less to do with the development or promotion of SoWMy and more to do with intra-country deficits that inhibited them from acting on the report’s suggestions: *contextual challenges.* Some, for example, noted that structural and geographical challenges within their countries, such as lack of necessary infrastructure in rural regions, limited their efforts. Additionally, many also referenced a lack of resources and funding as key obstacles.

### Theory of change

**Fig. 1:**
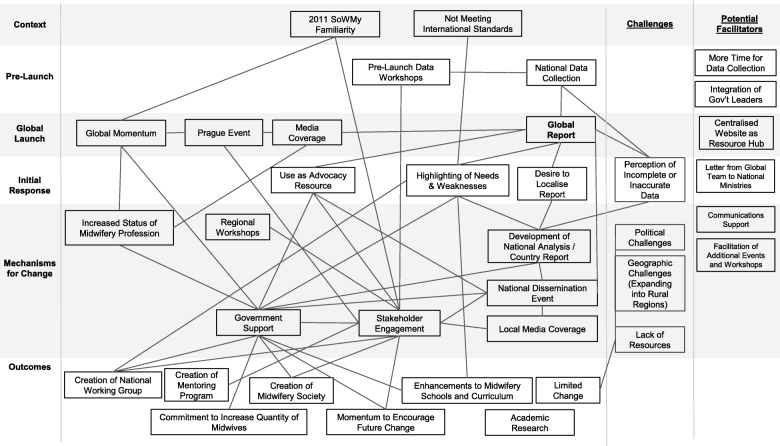
Theory of Change Reflecting Mechanisms for Policy Impact of SoWMy 2014 (links indicate ‘leading /contributing to’ if vertical, or ‘concurrent with’ if horizontal)

The policy impact framework allows for the creation of a Theory of Change (see Fig. 1), or a collection of pathways that highlight how the development of the global SoWMy report led to a range of outcomes on the national level. Below, we summarise how our findings draw connections between pre-launch activities, events surrounding the global launch of SoWMy, how the report was received on the national level, which mechanisms were used to generate change on the national level, and the outcomes that would eventually result. Challenges that impeded change, as well as the opportunities to facilitate greater change, are noted on the right of the model.

### Context

The data within the policy impact framework reveal that country-specific context prior to and during SoWMy’s launch often influenced the process by which the report would be utilised. Fertile contexts for change include not only those countries in which midwifery was already considered a high priority, but also in countries where reducing maternal morbidity and mortality was an ambition that had not yet been acted upon. Additionally, involvement with the 2011 SoWMy report generally enhanced receptivity of the 2014 version, but it was not necessary for a country to achieve timely outcomes.

### Pre-launch

Activities prior to the launch of the 2014 SoWMy – such as the data collection process and data workshops – began to shape perceptions of the eventual report in positive and negative ways.

For many, the data collection process necessarily involved governmental support and, thus, made it easier to gain political buy-in once the report’s findings were unveiled. In certain countries, however, frustrations with the timing of the data collection or the lack of clarity in how certain metrics were calculated prompted hesitancy among national organisations and government bodies.

### Global launch

The report was introduced at a global launch event in Prague as part of the International Confederation of Midwives. A key strategy of the launch was to garner media coverage of the report. As detailed in the *Results* section, the press release highlighting the report received more than 300 pickups, while stories featuring the report were published independently in several high-visibility outlets. Interview respondents cited this media coverage as important in providing national organisations with momentum and tools to prepare the mechanisms by which they would attempt to achieve progress within their country.

The data within the policy impact framework reveal that the launch and subsequent media coverage also helped to generate a global sense of momentum, which, according to many respondents, helped them to push efforts forward within their own countries. SoWMy added a sense of importance and urgency to the midwifery profession, prompting government officials to be more receptive to calls for policy or programmatic change, according to respondents.

### Initial response

The initial response of stakeholders reflects how they perceived and utilised the report after its global launch.

Most common was the use of SoWMy as an advocacy resource within countries. Stakeholders frequently used the report to make calls for policy and programmatic changes, or to bolster their case for acting on previously recommended changes.

As noted in the policy contribution framework, SoWMy was also influential in pinpointing particular country-specific weaknesses with respect to midwife practices. These findings sometimes led directly to outcomes, such as mechanisms to improve the quality of education related to midwifery training. More often, though, it simply served as an impetus for government officials and advocacy groups to begin the process of determining how to address the revealed weaknesses.

Finally, some countries also decided to create a localised version of the SoWMy report, as previously detailed. Thus, SoWMy provided an impetus for the local production of an evidence base.

### Mechanisms for change

As observed, acquiring government support and facilitating stakeholder interaction were near-universal steps in generating productive outcomes related to midwifery or maternal health. Regardless of the responses to SoWMy or tactics chosen within countries, gaining the support of these prominent groups appeared to be a necessary condition for change.

Interview respondents suggested that gaining government support and involving key stakeholders could be achieved in different ways. National events and workshops, though, appeared to be the most effective method of achieving government support, engaging stakeholders, and generating concrete policy or programmatic outcomes.

Finally, the increased status of midwifery profession, often cited a result of the global momentum that SoWMy generated, was both an outcome of the report and a propeller of additional progress.

## Discussion

We used a policy impact framework to evaluate the different impacts a global policy initiative had on different domains. Identifying the different types of impacts that occurred following this policy initiative allowed us to develop a workable theory of change and gain a greater understanding of the specific policy mechanisms at play among a diverse set of countries. These efforts serve two purposes: to identify potential facilitators for policymakers wishing to implement something similar in the future, and to identify mechanisms underpinning policy change. The policy impact framework employed within this study revealed that the SoWMy 2014 initiative led to positive, significant change in many participating countries and that, through various mechanisms, the global launch event and subsequent national events generated considerable media interest and political will, which contributed to political commitments and direct changes to midwifery provision and education. We did not identify any changes to behaviour, or to the physical environment. However, this may be because we were limited to desk-based data collection.

The impact framework identified several challenges. These fell into three groups: challenges associated with the difficulties individual countries were facing when attempting to address maternal morbidity; challenges associated with data collection and data analysis; and challenges associated with implementation and political acceptance of the report. Previous work identifies similar challenges for implementation of global health initiatives, including structural, sociocultural and value-related factors (29, 30).

Countries were starting with different levels of resources, as well as from different levels of acceptance that the reproductive and maternal health should be a priority and/or that expanded midwifery provision could be an effective solution to high levels of maternal and newborn mortality and morbidity. The wide variety of different starts points was highlighted within the report itself, which showed that some countries simply did not have enough SRMNH workers, whereas others had sufficient numbers but serious challenges relating to accessibility, acceptability and/or quality of the services provided by the workforce. Therefore, different countries needed the report to perform different functions, as evidenced by the variety of different outcomes recorded in this report, ranging from scaling up the numbers of midwives to updating the education curriculum.

Secondly, some respondents reported timing, resource and skill issues. In part, these comments probably reflect the challenges of trying to collect and analyse evidence on a very complex area. SoWMy 2014 attempted to move away from the reductionist ‘indicator’ model, towards something more innovative, robust and actionable, and this was necessarily more complex than what people have been used to. This challenge was noted by the Secretariat who provided support through publication of an advocacy toolkit, and through organisation of follow-up activities to address this issue – i.e. the helpdesk, regional workshops and production of working papers and journal articles focusing on specific parts of the findings.

Thirdly, political challenges around implementation and usage were described. While in a minority, some respondents described how the report was regarded as having low credibility for various reasons, which led to poor uptake or political support. However, in some cases, this perceived low reliability led to increased political leadership locally, which was a positive outcome if via a potentially challenging mechanism.

### Lessons for global policymakers

The theory of change seemed to indicate that policy impacts were increased where local contexts were positively inclined towards improving maternal health and midwifery services and where there was a concerted attempt among stakeholders to build off the momentum of the SoWMy launch to plan locally-relevant events, workshops and publications.

With this type of exercise, the provision of technical support to member states is crucial to address concerns related to the timing, complexity, and inclusion of the data-collection process. Additionally, further support from global policymakers like UNFPA, WHO, and others with post-launch implementation tactics and communication with national governments could aid national and local advocacy groups in achieving SoWMy’s recommendations.

### Lessons for national policymakers

This evaluation shows that active engagement of national policymakers in the process of producing a global report of this nature can bring about benefits in terms of providing a reliable evidence base on which to focus decisions and advocacy activities after the report’s launch. Engagement in follow-up dissemination activities can also build capacity at national level to use the most up-to-date techniques for data analysis.

### Lessons for researchers

The policy impact framework allowed us to identify key areas where changes were observed following the launch of a policy. While it is still not possible to directly attribute the changes to the policy in question, this information allows policymakers and researchers to identify multiple mechanisms of change which may be used to plan for maximum impact in similar future cases. Designing evaluation frameworks requires responsiveness to local conditions, and input from appropriate stakeholders [30].

As this was a complex data collection and analysis exercise, a useful lesson for researchers here is that provision of follow-up activities to help countries understand and make the most of the new material helps practitioners to grasp the challenge, and aids implementation. This is particularly important if, as in SoWMy, the techniques used for analysis are not straightforward for a non-technical audience to understand. Such activities need to be well publicised so that everyone knows what support is available.

The complexity of the analysis conducted for SoWMy 2014 required a long and detailed questionnaire, and this evaluation found that not everyone involved in the process could see the need for the level of detail requested. Clear communication from the research team of the reasons for particular data being requested is important to avoid respondents feeling as though they are being asked to do something unnecessarily complicated. In addition, we had limited resources to spend on data collection; it is possible that had we been able to conduct further interviews or observations, we would have identified further impacts such as ‘changes to behaviour’ or ‘changes to physical environment’.)

#### Limitations

Certain limitations prevented us from conducting a broader analysis of the impact of the SoWMy report.

It was not possible to contact the many hundreds of participants who had been involved at different times in the SoWMy process, including, importantly, the many thousands of midwives whose practice would have been directly affected; nor the women and children who hopefully benefitted from this initiative. We were only able to access a sample of these individuals, but attempted to design our sampling strategy to gather a representative range of responses. IN addition, we were only able to interview a limited number of policymakers who had been involved with, or acted upon the report. Additionally, language barriers prevented us from conducting an interview on one occasion and from collecting media and social media metrics that were not published in English or French. Contribution mapping should ideally be as comprehensive as possible, and our analysis would have been much richer had we been able to access these experiences.

Similarly, we were only able to collect respondents’ perceptions, rather than examining policy documents, or analysing midwifery / maternal mortality statistics. Although this was outside the resources available, it also seems unlikely that significant changes to maternal health (in many ways the ultimate outcome) would be visible in the short time since publication. Thus, we collected respondents’ attitudes and experiences to try and build a picture of the steps taken on the global and national levels following the release of SoWMy 2014.

Ultimately, this means that we were not able to present strength of evidence for each link in our theory of change, as we would have preferred. These links do not therefore indicate irrefutable causation, but rather that our evidence indicates that these factors contributed to each others’ existence and maintenance. In an ideal scenario, each link would be thoroughly empirically examined, which we were not able to do. However, we feel that this indicative theory should demonstrate the complexity of the mechanisms leading to policy change (particularly as it is likely missing links and factors), and more importantly shows the importance of taking a holistic approach to both creating, and evaluating policy change.

## Conclusion

Holistic policy evaluation can inform policymakers, practitioners and researchers about the mechanisms underpinning changes following policy implementation, and the factors influencing the direction and degree of these changes. As our findings demonstrate, SoWMy has served as an important and effective tool in encouraging changes relating to midwifery policies, programmes, practices, and understandings on the global and national levels. The data, as illustrated through our policy impact framework, point to specific areas of progress across a diverse range of countries.

Still, two sets of challenges prevented some countries from utilising SoWMy to its full extent. The first set is primarily process related, pointing to challenges that countries experienced during the data collection process. The second set relates to common structural and social roadblocks within different countries that prevented more significant changes from occurring.

Finally, the theory of change, which emerged from the policy impact framework, details the specific pathways and mechanisms by which progress (or a lack thereof) was made. Our findings draw connections between pre-launch activities, events surrounding the global launch of SoWMy, how the report was received on the national level, which mechanisms were used to generate change on the national level, challenges that impeded change, and the outcomes that would eventually result.

## Additional file

Additional file 1: Topic Guide. Questions and prompts used by interviewers to conduct semi-structured interviews with participants for the SoWMy study. (DOCX 14 kb)

## Abbreviations

SoWMy: State of the World’s Midwifery
SRMNH: Sexual, Reproductive, Maternal and Newborn Health
UN: United Nations
UNFPA: United Nations Population Fund
WHO: World Health Organisation
