# Supplementary material for: Assessing the policy and practice impact of an international policy initiative: the State of the World’s Midwifery 2014
Source: BMC Health Serv Res. 2018 Jun 27;18:499. doi: 10.1186/s12913-018-3294-4 (PMC6020347; doi:10.1186/s12913-018-3294-4)
Supplement: Supplementary file 1 — Topic Guide. Questions and prompts used by interviewers to conduct semi-structured interviews with participants for the SoWMy study. (DOCX 14 kb) [file 12913_2018_3294_MOESM1_ESM.docx]

**Topic guide for SoWMy interviews**

1. Can you tell me about yourself and your job role?
2. How did you get involved with SoWMy?
   1. 2011 / 2014?
   2. Who invited you?
   3. What was your role on it?
      1. Pre-launch: preparation and data collection?
      2. Post-launch?
   4. Tell me about your experiences
3. What would you say were the main aims of SoWMy?
   1. Globally
   2. For your country
4. What happened when it was launched?
   1. What were your main hopes and priorities for your country?
   2. What did you think the main challenges would be?
   3. What were you hoping would change (e..g numbers of midwifery posts, senior people’s attitudes, policy statements)
5. Now it is 18 months after the report’s launch, can you say that any of the changes that you desired were made?
   1. What other ways has SRMNH policy changed in your country since the launch of SoWMy?
   2. Do you think additional policy changes will occur in the near future?
   3. How did SOWMy report contribute to these changes?
      1. Did it contribute, or were things changing anyway?
   4. If there weren’t many changes, can you say why?
6. What types of factors helped the SOWMy report have an impact?
   1. Political support
   2. Media coverage
   3. Advocacy toolkit
   4. Follow-up workshops in Bangkok (Feb 2015) and/or Cairo (Nov 2015)
   5. Other mechanisms
7. What do you think the main challenges are for SRMNH in your country now?
   1. Have these changed since the launch of the report?
8. What would you have changed about how SoWMy was developed and implemented?
9. Any other comments on the report?
